# Negative Cellular Effects of Urban Particulate Matter on Human Keratinocytes Are Mediated by P38 MAPK and NF-κB-dependent Expression of TRPV 1

**DOI:** 10.3390/ijms19092660

**Published:** 2018-09-07

**Authors:** Kitae Kwon, See-Hyoung Park, Byung Seok Han, Sae Woong Oh, Seung Eun Lee, Ju Ah Yoo, Se Jung Park, Jangsoon Kim, Ji Woong Kim, Jae Youl Cho, Jongsung Lee

**Affiliations:** 1 Molecular Dermatology Laboratory, Department of Integrative Biotechnology, College of Biotechnology and Bioengineering, Sungkyunkwan University, Suwon City, Gyunggi Do 16419, Korea; nikon_40@naver.com (K.K.); hanzeeoo@naver.com (S.W.O.); sejs0701@naver.com (S.E.L.); wndk1929@skku.edu (J.A.Y.); tpwjd17@naver.com (S.J.P.); rovett@naver.com (J.K.); 2 Department of Bio and Chemical Engineering, Hongik University, Sejong City 300-16, Korea; imsesame@gmail.com; 3 AMI Cosmetic Co., Ltd., 19 Yanghwa-ro, Mapo-gu, Seoul 04026, Korea; bshan@skinami.co.kr (B.S.H.); jumpbook@skinami.co.kr (J.W.K.); 4 Molecular Immunology Laboratory, Department of Integrative Biotechnology, College of Biotechnology and Bioengineering, Sungkyunkwan University, Suwon City, Gyunggi Do 16419, Korea; 5 Biocosmetics Research Center, College of Biotechnology and Bioengineering, Sungkyunkwan University, Suwon City, Gyunggi Do 16419, Korea

**Keywords:** human keratinocytes, TRPV 1, MAPK, NF-κB, UPM, skin

## Abstract

Urban particulate matter (UPM) exerts negative effects on various human organs. Transient receptor potential vanilloid 1 (TRPV1) is a polymodal sensory transducer that can be activated by multiple noxious stimuli. This study aimed to explore the effects of the UPM 1648a on the expression of TRPV1, and its regulatory mechanisms in HaCaT cells. UPM enhanced TRPV 1 promoter-luciferase reporter activity. UPM also increased expression of the TRPV 1 gene as evidenced by increased mRNA and protein levels of TRPV 1. In addition, elucidation of the underlying mechanism behind the UPM-mediated effects on TRPV 1 expression revealed that UPM can upregulate expression of the TRPV1 gene by activating activator protein-1 (AP-1) and nuclear factor kappa B (NF-κB). The UPM treatment also altered Ca^2+^ influx and cell proliferation, as well as production of interleukin-8 (IL-8), tumor necrosis factor-α (TNF-α), and interleukin-1β (IL-1β). In addition, these UPM-induced effects were attenuated by SB203580 and ammonium pyrrolidinedithiocarbamate (PDTC). However, SP600125 and PD98059 did not alter the UPM-induced effects. Taken together, these findings indicate that UPM upregulates expression of the TRPV 1 gene, which is mediated by the p38 mitogen-activated protein kinase (MAPK) and NF-κB signaling pathways and suggest that UPM is a potential irritant that can induce skin processes such as aging and inflammatory responses.

## 1. Introduction

Urban particulate matter (UPM) has become an increasing risk factor to human health particularly in urban areas. A number of studies have shown that UPM affects several organs; including the cardiovascular and pulmonary systems [1,2,3,4], as well as several cell types including lung cells; macrophages; and epithelial cells [5,6,7]. In addition; epidemiological studies have shown that UPM exposure is correlated with several human diseases including bronchial asthma, chronic obstructive pulmonary disease, lung cancer, atherosclerosis, ischemic stroke, congestive heart failure, myocardial ischemia, and birth defects [8,9,10,11,12,13,14,15,16].

Transient receptor potential vanilloid 1 (TRPV 1), a polymodal sensory transducer, can be activated by multiple noxious stimuli, including pH, heat, endogenous lipid derivatives, and exogenous substances, which can cause pain, inflammation, and hyperalgesia [17]. TRPV 1, which functions as a calcium-permeable, non-selective cation channel, is highly expressed in primary sensory neurons and also widely expressed on various mesenchymal and epithelial human skin cell types such as mast cells, glial cells, bronchial epithelial cells, uroepithelial cells, and keratinocytes [18,19]. The activation of TRPV1 may be involved in proliferation, apoptosis, differentiation, and/or cytokine release. Phosphorylation of TRPV 1, leading to its enhanced function and membrane translocation, is a potential mechanism underlying inflammation-mediated hyperalgesia [20,21,22].

UPM can penetrate the human body through the oral and respiratory routes. Skin is another common pathway through which chemicals or xenobiotics enter the body. Alterations in the structure and function of the skin barrier can lead to various skin diseases [23]. Topical exposure to UPM has been reported to induce allergic skin reactions, skin aging, and delayed wound healing [24,25,26]. However, there have been fewer investigations thus far regarding the impacts of UPM on the skin compared to those pertaining to the cardiovascular and respiratory systems. In particular, the effects of UPM on the expression and function of TRPV 1 have not been reported. Therefore, the purpose of this study was to investigate the effects of the UPM 1648a on the expression of TRPV 1 and its regulatory mechanisms in human keratinocytes.

## 2. Results

### 2.1. Urban Particulate Matter Upregulates TRPV 1 Gene Expression in Human Keratinocytes

**Figure 1:**
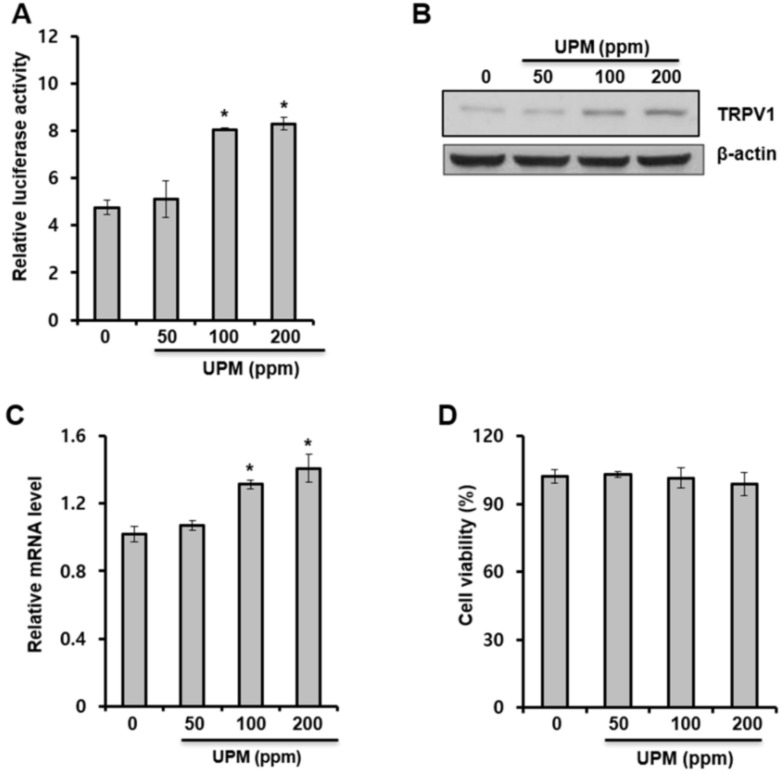
Urban particulate matter upregulates transient receptor potential vanilloid 1 (TRPV 1) gene expression in human keratinocytes. (**A**) HaCaT cells were transfected with the TRPV 1-Luc reporter along with a Renilla-luciferase expression vector that was driven by a thymidine-kinase promoter using DharmaFECT^®^ Duo transfection reagent according to the manufacturer’s protocols. After 24 h, the cells were incubated in the presence of the indicated concentrations of UPM for 14 h. The cells were subjected to luciferase reporter assay. *p* < 0.05 vs. control. (**B**–**D**) HaCaT cells were incubated with the indicated concentrations of UPM for two days. Western blot analysis for TRPV 1 was performed on the cell lysates (**B**), and the mRNA levels of the indicated genes were measured using quantitative real-time PCR (qRT-PCR) analysis (**C**). The expressed results are relative to the untreated cells after normalization against the GAPDH level. In addition, viability of cells exposed to urban particulate matter (UPM) was measured using WST-1 assay (**D**). * *p* < 0.05 vs. control. The results were confirmed using four independent experiments. Each experiment was conducted in duplicate. The data are expressed as the mean ± SD.

In this study, luciferase reporter assays, Western blot analysis, and quantitative real-time PCR (qRT-PCR) analysis were performed to investigate the effects of UPM on the expression of the TRPV 1 gene in HaCaT cells. As shown in Figure 1A, UPM increased TRPV 1-promoter reporter activity. In addition, the protein and mRNA levels of TRPV 1 were increased by UPM treatment (Figure 1B, Figure 1C, respectively). In addition, we found that UPM had no cytotoxic effects at the treated concentrations (Figure 1D). These data indicate that UPM increases the expression of the TRPV1 gene and suggest that UPM could mediate TRPV 1-induced effects.

### 2.2. Urban Particulate Matter Activates AP-1-, CRE-, and NF-κB-Signaling

**Figure 2:**
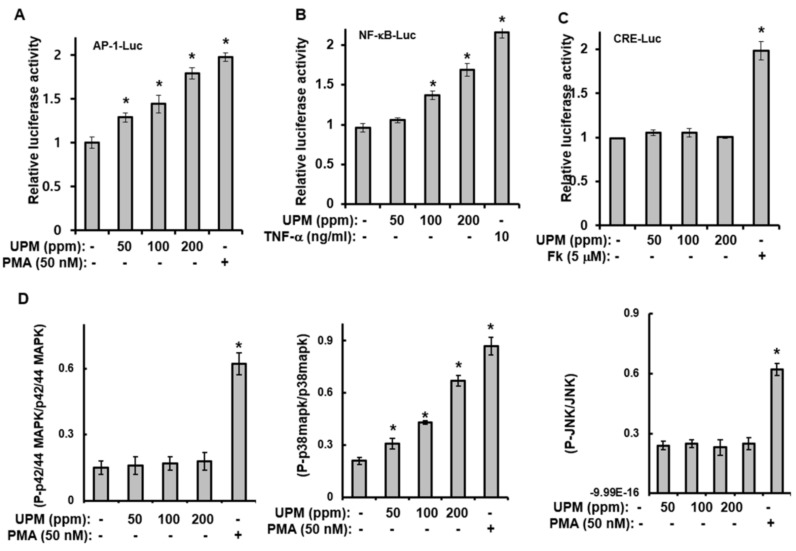
Urban particulate matter activates activator protein-1 (AP-1)-, cAMP response element (CRE)-, and nuclear factor kappa B (NF-κB)-signaling. HaCaT cells were transfected with the AP-Luc (**A**), NF-κB-Luc (**B**), or CRE-Luc (**C**) reporter along with a Renilla-luciferase expression vector driven by a thymidine-kinase promoter using DharmaFECT^®^ Duo transfection reagent according to the manufacturer’s protocols. After 24 h, the cells were incubated in the presence of the indicated concentrations of UPM for 14 h. The cells were then harvested, lysed, and assayed. (**D**) HaCaT cells were treated with the indicated concentrations of UPM for 1 h. The cell lysates were then analyzed using the Multi-Target Sandwich ELISA Kit. The results were verified using three independent experiments, each of which was conducted in duplicate. * *p* < 0.05 vs. control. The data are expressed as the mean ± SD. PMA, Phorbol 12-myristate 13-acetate; Fk, forskolin; TNF-α, tumor necrosis factor-α.

To elucidate the underlying mechanism behind the UPM-mediated effects on TRPV 1 expression, luciferase-reporter assays for AP-1, cAMP response element (CRE), and NF-κB, as well as Western blot analyses, were performed. The luciferase-reporter assays showed that although UPM increased the activity of the AP-1- and NF-κB-luciferase reporter (Figure 2A,B), CRE-reporter activity was not affected by UPM treatment (Figure 2C). In addition, treatment with UPM increased the phosphorylation of p38 mitogen-activated protein kinase (MAPK); however, JNK and p42/44 MAPK were not activated by the UPM treatment (Figure 2D). Taken together, these results suggest that UPM can increase the expression of the TRPV 1 gene through activation of the p38 MAPK and NF-κB signaling pathways.

### 2.3. UPM-induced Expression of TRPV 1 Is Mediated through Activation of p38 MAPK and NF-κB

**Figure 3:**
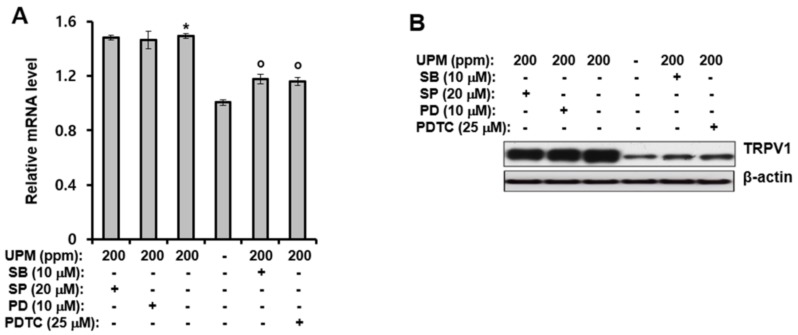
UPM-induced expression of TRPV 1 is mediated through activation of p38 mitogen-activated protein kinase (MAPK) and NF-κB. HaCaT cells were treated with UPM (200 ppm) and then incubated for two days in the presence of the indicated concentration of MAPK and NF-κB inhibitors. (**A**) Total RNA was isolated from the cells, and the mRNA levels of the indicated genes were measured using real-time quantitative RT-PCR. The expressed results are relative to the untreated cells after normalization against the GAPDH level. * *p* <0.05 vs. the untreated control, ^o^ *p* <0.05 vs. the UPM-treated control. The results were confirmed using four independent experiments. Each experiment was conducted in duplicate. The data are expressed as the mean ± SD. (**B**) The cell lysates were subjected to Western blot analysis for TRPV 1. UPM, urban particulate matter; SB, SB203580; SP, SP600125; PD, PD98059; PDTC, ammonium pyrrolidinedithiocarbamate.

The previous section revealed that UPM can increase TRPV 1 gene expression through activation of p38 MAPK and NF-κB. Therefore, the effects of inhibitors of MAPKs and NF-κB on the UPM-induced expression of TRPV 1 were examined. As shown in Figure 3A,B, treatment of HaCaT cells with SB203580 (a p38 MAPK inhibitor), and ammonium pyrrolidinedithiocarbamate (PDTC, an NF-κB inhibitor), significantly reduced the mRNA and protein levels of TRPV 1 induced by UPM. However, SP600125 (a JNK inhibitor) and PD98059 (a p42/44 MAPK inhibitor) did not alter the UPM-indced effects. Collectively, these results suggest that UPM can increase expression of TRPV 1 by activating NF-κB and p38 MAPK.

### 2.4. UPM Increases Ca^2+^Influx and The Production of Pro-inflammatory Cytokines

**Figure 4:**
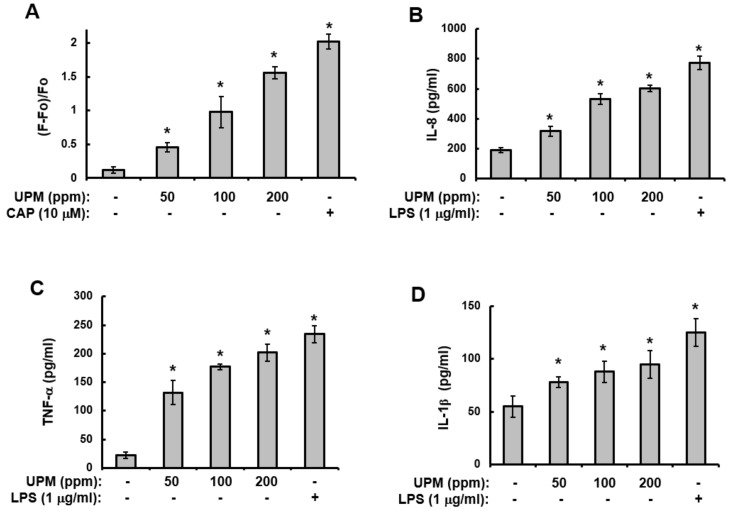
UPM increases Ca^2+^ influx and the production of pro-inflammatory cytokines. (**A**) HaCaT cells were loaded with Fura2-AM for 60 min and then washed with Krebs-HEPES buffer for the [Ca^2+^]_i_ measurement. The [Ca^2+^]_i_ was measured fluorometrically every 5 s for 100 sec after the addition of 200 ppm UPM, as described in the Materials and Methods (Section 4). The curve shown is representative of three independent experiments. (**B**–**D**) HaCaT cells were treated with the indicated concentrations of UPM for two days. The supernatants were then analyzed using the Multi-Target Sandwich ELISA Kits for interleukin-8 (IL-8) (**B**), TNF-α (**C**), and IL-1β (**D**). The results were verified using three independent experiments, each of which was conducted in duplicate. * *p* < 0.05 vs. control. The data are expressed as the mean ± S.D. UPM, urban particulate matter; LPS, lipopolysaccharide; CAP, capsaicin.

The previous section revealed that UPM can enhance the expression levels of the TRPV 1 gene through the activation of NF-κB and p38 MAPK. In addition, it has been reported that Ca^2+^ influx and the production of pro-inflammatory cytokines are downstream events of TRPV 1 [22,27]. Therefore, to further determine the role of UPM in the expression of TRPV 1, assays for Ca^2+^ influx and the production of pro-inflammatory cytokines were performed. As shown in Figure 4, treatment with UPM increased Ca^2+^ influx (Figure 4A) and the production of interleukin-8 (IL-8) (Figure 4B), TNF-α (Figure 4C), and IL-1β (Figure 4D) in a concentration-dependent manner. These results indicate that UPM activates TRPV 1-mediated events.

### 2.5. UPM-induced Increases in Ca^2+^Influx and The Production of Pro-inflammatory Cytokines are Mediated Through Activation of p38 MAPK and NF-κB

**Figure 5:**
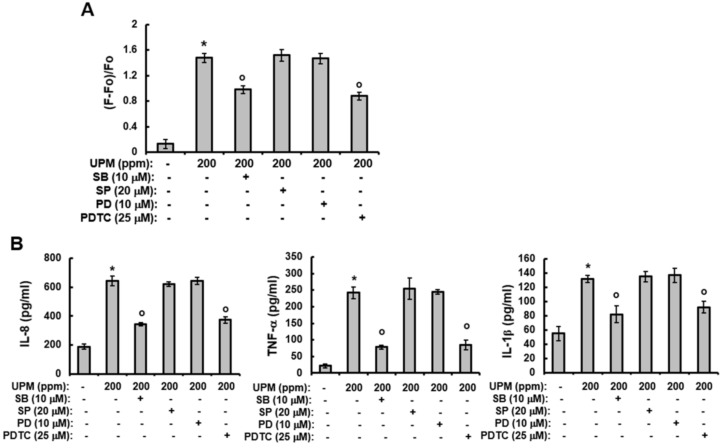
UPM-induced increases in Ca^2+^ influx and production of pro-inflammatory cytokines is mediated through activation of p38 MAPK and NF-κB. (**A**) HaCaT cells were loaded with Fura2-AM for 60 min and then washed with Krebs-HEPES buffer for the [Ca^2+^]_i_ measurement. The [Ca^2+^]_i_ was measured fluorometrically every 5 s for 100 sec after the addition of 200 ppm UPM as described in the Materials and Methods. The curve shown is representative of three independent experiments. (**B**) HaCaT cells were treated with UPM (200 ppm) and then incubated for two days in the presence of the indicated concentration of inhibitors. The supernatants were then analyzed using the Multi-Target Sandwich ELISA Kits for IL-8, TNF-α, and IL-1β. The results were verified using three independent experiments, each of which was conducted in duplicate. * *p* < 0.05 vs. the untreated control, ^o^ *p* < 0.05 vs. the UPM-treated control. The data are expressed as the mean ± SD. UPM, urban particulate matter; SB, SB203580; SP, SP600125; PD, PD98059; PDTC, ammonium pyrrolidinedithiocarbamate.

The above results indicate that UPM can increase the expression of TRPV 1 and mediate TRPV 1-induced events and that the UPM-induced expression of TRPV 1 is mediated through the activation of p38 MAPK and NF-κB. Therefore, we examined the effects of MAPK inhibitors on the increased influx of Ca^2+^ and production of pro-inflammatory cytokines induced by UPM. As with TRPV 1 expression, although SB203580 and PDTC significantly attenuated both the UPM-induced effects on Ca^2+^ influx (Figure 5A) and the production of pro-inflammatory cytokines (Figure 5B), SP600125 and PD98059 did not alter the UPM-induced effects. Collectively, these results indicate that UPM activates the expression of TRPV 1 and TRPV 1-mediated signaling by activating p38 MAPK and NF-κB. 

### 2.6. Effects of UPM On Cell Proliferation and ROS Production

**Figure 6:**
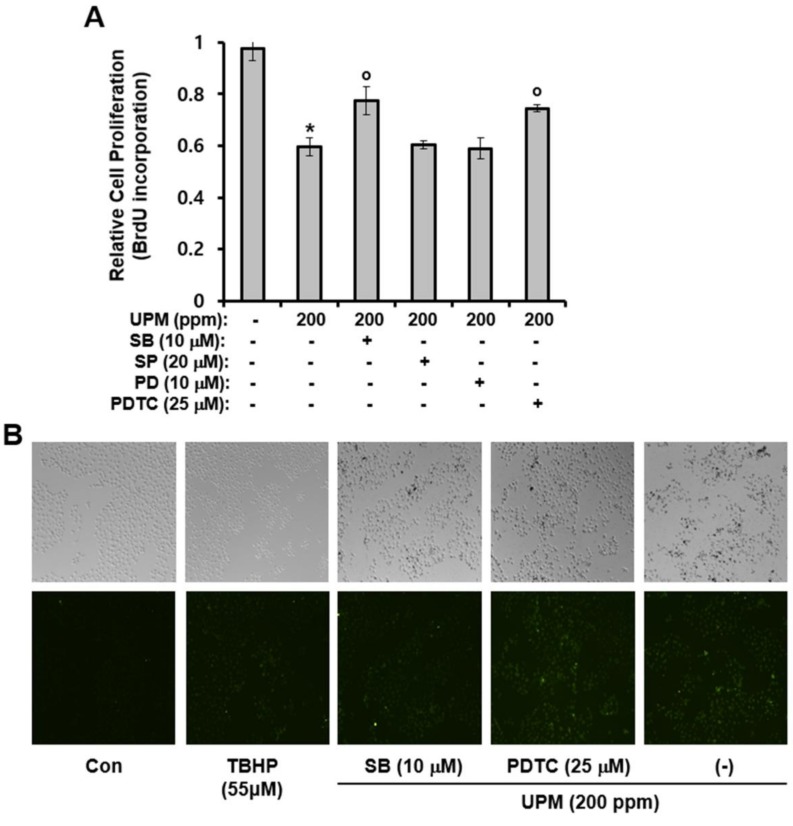
UPM-induced effects on cell proliferation and ROS production in HaCaT cells. (**A**) HaCaT cells were treated with the indicated concentrations of UPM and then incubated for two days in the presence of the indicated concentration of inhibitors. The results were verified using three independent experiments, each of which was conducted in duplicate. * *p* < 0.05 vs. the untreated control, ^o^ *p* < 0.05 vs. the UPM-treated control. The data are expressed as the mean ± SD. (**B**) HaCaT cells were treated with UPM (200 ppm) and then incubated for two days in the presence of the indicated concentration of inhibitors. ROS production was evaluated using 2’,7’-dichlorofluorescein diacetate. UPM, urban particulate matter; SB, SB203580; SP, SP600125; PD, PD98059; PDTC, ammonium pyrrolidinedithiocarbamate; TBHP, tert-Butyl hydroperoxide.

In this study, cell-proliferation and ROS production assays were performed to investigate the effect of UPM on proliferation of HaCaT cells and cellular levels of ROS. As shown in Figure 6A,B, while proliferation was reduced by UPM treatment, UPM increased production of ROS. In addition, as with Ca^2+^ influx and the production of pro-inflammatory cytokines, SB203580 and PDTC significantly attenuated the UPM-induced effects on cell-proliferation (Figure 6A). However, SP600125 and PD98059 did not alter the UPM-induced effect on cell-proliferation. Furthermore, the UPM-induced production of ROS was not altered by SB203580 and PDTC (Figure 6B). Collectively, these results suggest that while UPM-induced retardation of cell proliferation may be affected by p38 MAPK and NF-κB–dependent expression of TRPV 1, UPM-induced production of ROS may not be dependent on TRPV 1 expression. 

### 2.7. UPM Affected Morphology of the Reconstructed Epidermis and Reduced Expression of Proliferating Cell Nuclear Antigen (PCNA) Gene and Filaggrin Gene

**Figure 7:**
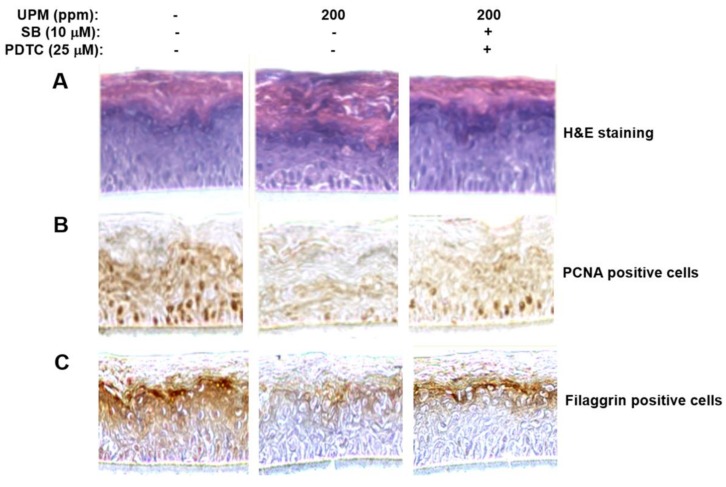
Effects of UPM on structure of reconstructed human epidermis, and expression of PCNA and filaggrin genes in the reconstructed epidermis. (**A**) Reconstructed epidermis was incubated in medium containing 200 ppm of UPM for 96 h in the presence of SB203580 and PDTC and then subjected to hemoxylin and eosin (H and E) staining and immunohistochemical staining for PCNA (**B**) and filaggrin (**C**). UPM, urban particulate matter; SB, SB203580; PDTC, ammonium pyrrolidinedithiocarbamate.

**Figure 8:**
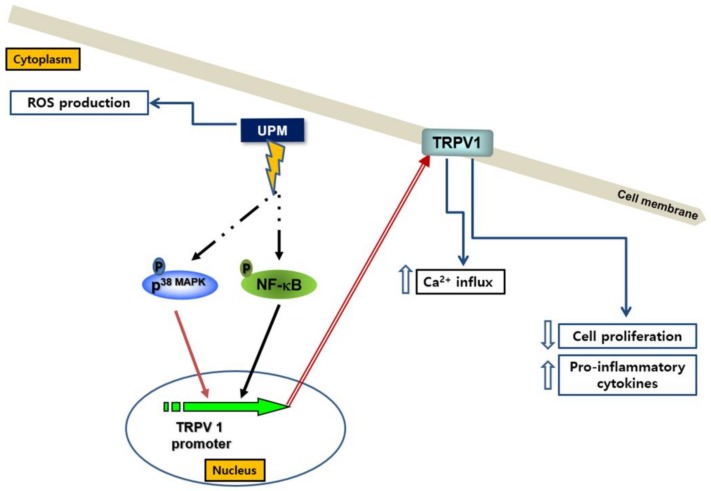
Mechanisms involved in the effect of UPM on TRPV 1-induced signaling. UPM induces the activation of p38 MAPK and NF-κB, which sequentially upregulate expression of TRPV1 gene. TRPV1 contributes to the increase of calcium influx, production of cytokines, as well as the decrease of cell proliferation. However, it is not involved in the production of ROS.

Using human reconstructed epidermis, we investigated effects of UPM on the structure of the epidermis and expression of proliferation-related and differentiation-related genes such as PCNA and filaggrin. As shown in Figure 7A, hemoxlinand eosin staining showed that UPM altered the structure and morphology of the reconstructed epidermis. In addition, UPM reduced expression of *PCNA* and filaggrin genes, which are one of proliferation-related genes, and one of differentiation-related genes, respectively (Figure 7B,C). Furthermore, we found that SB203580 and PDTC significantly attenuated both the UPM-induced effects on the structure, and morphology of the reconstructed epidermis (Figure 7A), and expression of PCNA and filaggrin genes (Figure 7B,C). Collectively, these results suggest that UPM exerts negative effects on skin structure, proliferation, and differentiation by downregulating TRPV 1. The possible action mechanisms involved in the effect of UPM on TRPV1-mediated signaling are shown in Figure 8.

## 3. Discussion

UPM can induce abnormal skin states such as allergic reactions, aging, and delayed wound healing [24,25,26]. In addition, TRPV 1 can be activated by multiple harmful stimuli, including pain, inflammation, and hyperalgesia, and this activation can mediate their effects [17]. However, until now, the relationship between UPM and TRPV 1 has not been clearly elucidated. Specifically, the involvement of TRPV 1 in UPM’s effects on the skin has not been directly shown. Therefore, in this study, we demonstrated that UPM-treatment can increase the expression of TRPV 1 through activating NF-κB and p38 MAPK, thus increasing Ca^2+^ influx and the production of pro-inflammatory cytokines. In addition, the activating effects of UPM on the expression of the TRPV 1 gene may be related with UPM-induced skin sensitivity.

The expression of TRPV 1 plays an important role in the regulation of various symptoms—such as pain, inflammation, hyperalgesia, and skin irritation [17,18,19]. However, the mechanisms of TRPV 1 gene expression have not been fully investigated. In particular, the involvement of UPM on the expression of TRPV 1 has not been reported. In this study, we demonstrated that UPM can upregulate the expression of TRPV 1 in HaCaT cells. In addition, we elucidated the underlying mechanism behind the UPM-mediated effects on TRPV 1 expression. The results showed that the UPM-induced upregulation of TRPV 1 was mediated through activation of AP-l and NF-κB. Among the MAPKs, only p38 MAPK was involved in the expression of TRPV 1, and p42/44 MAPK and JNK had no effects on the expression. These results indicate that UPM can upregulate expression of TRPV 1 by selectively inducing the activation of NF-κB and p38 MAPK.

Ca^2+^ influx and the expression of pro-inflammatory cytokines are important downstream events that are regulated by TRPV 1 [9,27], the expression of which is in turn upregulated by UPM treatment. In this study, the level of Ca^2+^ influx and the expression levels of IL-8, TNF-α, and IL-1β were increased by UPM treatment. These results suggest that UPM can increase Ca^2+^ influx, and the levels of pro-inflammatory cytokines by upregulating the expression of TRPV 1, consequently inducing abnormal skin symptoms.

UPM exerts numerous effects on cells and tissues [1,2,3,4,5,6,7]. It has been reported that exposure to UPM correlates with pulmonary dysfunction, cardiovascular disease, and hepatic fibrogenesis [28,29,30]. However, most of the studies on the health-related adverse effects of UPM have focused on cardiovascular and respiratory dysfunction. Currently, the skin is becoming a major target of research on UPM-induced toxic effects because it constitutes the outermost barrier that comes into direct contact with air pollutants [31,32]. This study showed that UPM can increase the expression of TRPV 1 in human keratinocytes by selectively activating NF-κB and p38 MAPK. This suggests that molecules that downregulate expression of TRPV 1 could antagonize the UPM-induced adverse effects to the skin and that TRPV 1 might be a good therapeutic target to improve UPM-induced skin diseases.

In conclusion, the results of this study indicate that UPM can exert damaging effects on the skin by increasing the expression of TRPV 1 through activating NF-κB and p38 MAPK, thereby increasing the production of pro-inflammatory cytokines.

## 4. Materials and Methods 

### 4.1. Materials

Dulbecco’s modified Eagle’s medium (DMEM) was obtained from Santa Cruz Biotechnology, Inc. (Santa Cruz, CA, USA). PD98059, SP600125, and SB203580 were purchased from Calbiochem (La Jolla, CA, USA). Ammonium pyrrolidinedithiocarbamate (PDTC), forskolin (Fk), capsaicin, lipopolysaccharide (LPS), and Phorbol 12-myristate 13-acetate (PMA) were obtained from Sigma Chemical Co. (St. Louis, MO, USA). The protease inhibitor cocktail was purchased from Roche (Indianapolis, IN, USA). Urban particulate matter (UPM) (the standard reference material, 1648a) was supplied by National Institute of Standards and Technology (NIST) (Gaithersburg, MD, USA). The composition of UPM was shown in Table 1. UPM was suspended in deionized water at a concentration of 50 mg/mL and stored at −20 °C until used. UPM stock solution was sonicated for 5 min using Bioruptor sonicator (Cosmobio, Tokyo, Japan) immediately before treatment. Working solution of UPM was prepared in 500 μL DMEM and added to the cultured cells.

### 4.2. Culture of HaCaT cells

HaCaT cells (a human keratinocyte cell line) were obtained from ATCC (Manassas, VA, USA). The cells were cultured in Dulbecco’s modified Eagle’s medium (DMEM) supplemented with 10% fetal bovine serum (FBS) and 1% solution containing penicillin and streptomycin in a humidified, 5% CO₂ atmosphere at 37 °C. The cells were then expanded through ten passages in DMEM. The medium was changed every three days until the cells reached 70% confluence, at which time they were passaged.

### 4.3. Assay for Cytotoxicity of UPM

The cytotoxicity of UPM in HaCaT cells was measured by WST-1 method to determine an optimal concentration for the study. HaCaT cells were seeded in a 96-well plate at a density of 2 × 10^4^ cells per well. Cells were treated with 500 μL of 0, 50, 100, and 200 ppm of UPM for two days. After incubation with UPM, as per the manufacturer’s instructions, 40 μL of WST-1 (TaKaRa Bio Inc., Shiga, Japan) was added to each well. Cells were incubated for 1 h and the medium was collected in micro-centrifuge tubes, and spun-down to precipitate the UPM particles. Subsequently, 150 μL of collected medium was transferred to a 96-well plate for measuring optical density. Cytotoxicity was quantified by measuring the absorbance at 440 and 690 nm using a microplate reader (VERSAmax, Molecular Devices, Sunnyvale, CA, USA).

### 4.4. BrdU-Incorporation Assay

HaCaT cells were grown in serum-free culture medium, with or without the indicated concentrations of UPM, for two days. After two days, the cell proliferation was determined using the BrdU Cell Proliferation Assay Kit (Cell Signaling Technology, Danvers, MA, USA) according to the manufacturer’s instructions.

### 4.5. Reverse Transcription

HaCaT cells were grown in serum-free culture medium, with or without the indicated concentrations of UPM, for two days, and the total RNA was extracted using TRIzol reagent (Invitrogen, Carlsbad, CA, USA) and then purified using the RNeasy Mini Kit (Qiagen, Hilden, Germany) according to the manufacturer’s instructions. The purified RNA was then treated with DNase (Ambion, Austin, TX, USA) and analyzed using an Agilent Bioanalyzer (Agilent Technologies, Waldbronn, Germany) and a NanoDrop 8000 spectrophotometer (Thermo Scientific, Schwerte, Germany) to measure the RNA concentration, integrity, and purity. RNA samples with the RNA purity (A_260_/A_280_ = 1.8 to 2.0) and RNA-integrity number (RIN, ≧8.0) were used for studies. The purified RNA (1 μg) was reverse-transcribed in a 20-μL reaction mixture using the RevertAid^TM^ First Strand cDNA Synthesis Kit (Fermentas, Burlington, ON, Canada) and a Bio-Rad PTC-200 DNA Engine thermal cycler (Bio-Rad, Hercules, CA, USA) according to the manufacturer’s instructions. In brief, oligo (dT) primers and the RNA samples were mixed together and denatured at 70 °C for 10 min. The reaction mixtures were then immediately placed on ice for at least 1 min. RNase inhibitor was added to the transcription mixture, and the mixture was then incubated at 37 °C for 5 min. The first-strand cDNA synthesis was initiated after the addition of Moloney Murine Leukemia Virus (M-MuLV) Reverse Transcriptase (Thermo Fisher Scientific, San Jose, CA, USA), and the reverse transcription reaction was further conducted at 42 °C for 1 h. Finally, the reverse transcriptase enzyme was inactivated by incubating at 70 °C for 10 min. The reaction was conducted in triplicate to reduce any differences in reverse-transcription efficiency. The cDNAs were stored at −80 °C and then diluted 5-fold with RNase-free water prior to use as a template in the quantitative real-time PCR (qRT-PCR) analysis.

### 4.6. Quantitative Real-time-PCR (qRT-PCR) Analysis

The quantitative real-time PCR (qRT-PCR) analysis was performed using an ABI7900HT Real-Time PCR system (Applied Biosystems). The TaqMan RT-PCR reagents (the probes and primers) were obtained from Applied Biosystems. Predesigned and optimized Assays-on-Demand kits (Applied Biosystems, GAPDH:Hs00266705_g1, TRPV 1:Hs00218912_m1) were used for the TaqMan analysis. The reaction parameters were as follows: 2 min at 50 °C, 30 min at 60 °C, and 5 min at 95 °C, followed by 45 cycles of 20 s at 94 °C for denaturation, and 1 min at 60 °C for annealing and extension. All of the measurements were conducted in either duplicate or triplicate. ABI sequence-detection software, version 2.0 (Applied Biosystems) was used for data analysis. A relative quantitation was performed using GAPDH as a reference gene and validated using Norm Finder software. Because all of the assays used were optimized for PCR efficiency by the manufacturer, the mRNA-expression levels were determined using the delta-Ct method.

### 4.7. Measurement of Intracellular Level of Ca^2+^

Fura2-AM (a calcium-sensitive fluorescent dye) was used for measurement of [Ca^2+^]_i_. HaCaT cells were washed with Krebs-HEPES buffer (120 mM NaCl, 5.4 mM KCl, 1.5 mM CaCl_2_, 1 mM NaH_2_PO_4_, and 10 mM HEPES (pH 7.4)), and subsequently incubated with Fura2-AM at 37 °C for 1 h. The cells were then washed with Krebs-HEPES buffer and resuspended at 1 × 10^6^ cells/mL in the same buffer. Three ml of the cell suspension was placed in a quartz cuvette in a luminescence spectrometer (Perkin-Elmer, LS50B). The results are plotted as fluorescence changes relative to the untreated control expressed as (F-Fo)/Fo, where F and Fo are the fluorescence intensity of the UPM-treated samples and the untreated control, respectively.

### 4.8. Assay for Luciferase-Reporter Activity

To measure activities of the TRPV 1, AP-1-, NF-κB-, and CRE-promoter, HaCaT cells were transfected with TRPV 1-Luc (GeneCopoeia, Inc., Rockville, MD, USA), AP-1 (Stratagene, La Jolla, CA, USA), NF-κB (Stratagene) or CRE-Luc (Stratagene) along with 1 μg of the Renilla-luciferase expression vector driven by a thymidine-kinase promoter (Promega, Madison, WI, USA) (internal standard) using DharmaFECT^®^ Duo transfection reagent (Thermo Fisher Scientific, Inc., Waltham, MA, USA) according to the manufacturer’s instructions. At 24 h post-transfection, the cells were exposed to the indicated concentrations of UPM (NIST, Gaithersburg, MD, USA) in the presence or absence of forskolin (Tocris, Bristol, UK), phorbolmyristate acetate (PMA) (Sigma–Aldrich, St. Louis, MO, USA), tumor-necrosis factor-α (TNF-α) (Sigma–Aldrich), PD98059 (Cell Signaling Technology, Beverly, MA, USA), SB203580 (Cell Signaling Technology), SP600125 (Cell Signaling Technology), pyrrolidinedithiocarbamate (PDTC) (Sigma–Aldrich), or N-(2-[p-Bromocinnamylamino]ethyl)-5-isoquinolinesulfonamide (H89) (Sigma–Aldrich) for 24 h. The cells were then harvested, lysed, and centrifuged. Next, the supernatants were assayed for luciferase activity using the Dual Luciferase Assay system (Promega) and an LB953 luminometer (Berthold, Bad Wildbad, Germany). The activities are expressed as the ratio of the AP-1-, TRPV1-, NF-κB-, or CRE-dependent firefly-luciferase activity to the thymidine-kinase Renilla-luciferase activity that served as a control (i.e., % control). The results were confirmed with eight independent transfections.

### 4.9. Immunoblotting

The HaCaT cells were exposed to the indicated concentrations of UPM for the indicated time under serum-free conditions. The cells were washed twice with cold PBS (Sigma–Aldrich) and then lysed with 150 μL of sample buffer (100 mM Tris-HCl, pH 6.8, Sigma–Aldrich) with 10% glycerol (Sigma–Aldrich), 4% sodium dodecyl sulfate (SDS) (Sigma–Aldrich, St. Louis, MO, USA), 1% bromophenol blue (Sigma–Aldrich), and 10% β-mercaptoethanol (Sigma–Aldrich). The samples were resolved using sodium dodecyl sulfate-polyacrylamide gel electrophoresis (SDS-PAGE) and transferred to Immobilon-P PVDF membranes (Millipore Corporation, Bedford, MA, USA). The membranes were incubated overnight at 4 °C with an anti-TRPV 1 antibody (Invitrogen, Carlsbad, CA, USA) and an anti-β-actin antibody (Sigma–Aldrich). The membranes were subsequently washed three times with Tris-buffered saline containing Tween-20 (TBST; Sigma–Aldrich), probed with a horseradish peroxidase-conjugated secondary antibody (Sigma–Aldrich), and developed using an enhanced chemiluminescence (ECL) Western-blot detection system (Amersham Biosciences).

### 4.10. Analysisof MAPK-Phosphorylation

The levels of JNK, p38 MAPK, phospho-SAPK/JNK (Thr183/Tyr185), and phospho-p38 MAPK (Thr180/Tyr182) were determined using the PathScan InflammationMulti-Target Sandwich ELISA Kit (Cell Signaling Technology) according to the manufacturer’s instructions. The levels of p42/44 MAPK expression and phospho-p42/44 MAPK (Thr202/Tyr204) were determined using the PathScan Cell Growth Multi-Target Sandwich ELISA Kit (Cell Signaling Technology) according to the manufacturer’s protocols.

### 4.11. ELISA

HaCaT cells were exposed to the indicated concentrations of UPM for the indicated time under serum-free conditions. After the incubation, the concentrations of IL-8, TNF-α, and IL-1β in the culture supernatant were determined using an ELISA kit for IL-8 and TNF-α (ENZO Life Sciences International, Inc., Plymouth Meeting, PA, USA) and an ELISA kit for IL-1β (Genzyme, Minneapolis, MN, USA). The culture supernatants were added to 96-well plates. Alkaline phosphatase-conjugated IL-8, IL-1β, or TNF-α and their corresponding antibodies were added to the sample wells and incubated at room temperature for 2 h. The sample wells were then washed with PBS followed by the addition of a p-nitrophenyl phosphate (pNpp)-substrate solution. The samples were incubated at room temperature for 1 h, and their absorbance values were determined according to the manufacturer’s instructions.

### 4.12. Immunohistochemical Staining

Reconstructed Human Epidermis (Episkin^TM^, France) consisting of normal human-derived epidermal keratinocytes was cultured to form a multilayer and to become highly differentiated. Reconstructed epidermis was incubated in medium containing 200 ppm of UPM for 96 h. The reconstructed epidermis was immersed in a 10% buffered formaldehyde using ethanol, embedded in paraffin wax, and sliced at a thickness of 3 mm. The samples were stained by hemoxylin and eosin (H and E) and imaged under light microscopy using a Zeiss Axiovert 25 microscope (Zeiss, Oberkochen, Germany). Immunohistochemistry was performed using anti-proliferating cell nuclear antigen (PCNA) and anti-filaggrin (abcam, Cambridge, MA, USA) antibodies. The stained epidermis with PCNA and filaggrin was visualized by light microscopy.

### 4.13. Measurement of ROS Level

Intracellular ROS levels were detected using 2′,7′-dichlorofluorescin diacetate (DCF-DA; Sigma). HaCaT were incubated with 20 µM DCF-DA for 15 min before visualization under fluorescence microscopy. The fluorescence signals were obtained using a Nikon Eclipse Ti inverted microscope with a CCD-cooled camera (Nikon, Tokyo, Japan).

### 4.14. Statistical Analysis

All of the data are expressed as the mean ± SD. Comparisons between the control and the treated groups were performed using one-way analysis of variance (ANOVA) followed by Tukey’s multiple-comparison test using GraphPad Prism, version 5.0 (GraphPad, La Jolla, CA, USA). Statistical significance was considered when the *p*-value is less than 0.05.

## Figures and Tables

**Table 1:** Composition of UPM.

	Component	Concentration	Component	Concentration
Elements	Aluminum (Al)	3.43 ± 0.13 (%)	Iron (Fe)	3.92 ± 0.21 (%)
	Bromine (Br)	502 ± 10 (mg/kg)	Manganese (Mn)	790 ± 44 (mg/kg)
	Calcium (Ca)	5.84 ± 0.19 (%)	Sodium (Na)	4240 ± 60 (mg/kg)
	Chlorine (Cl)	4543 ± 47 (mg/kg)	Sulfur (S)	5.51 ± 0.36 (%)
	Chromium (Cr)	402 ± 13 (mg/kg)	Titanium (Ti)	4021 ± 86 (mg/kg)
	Copper (Cu)	610 ± 70 (mg/kg)	Zinc (Zn)	4800 ± 270 (mg/kg)
Polycyclic aromatic hydrocarbons (PAHs)	Phenanthrene	4.86 ± 0.17 (mg/kg)	Chrysene	6.12 ± 0.06 (mg/kg)
	Fluoranthene	8.07 ± 0.14 (mg/kg)	Benzo[e]pyrene	4.85 ± 0.07 (mg/kg)
	Pyrene	5.88 ± 0.07 (mg/kg)	Benzo[ghi] perylene	5.00 ± 0.18 (mg/kg)
	Benz[a] anthracene	2.71 ± 0.15 (mg/kg)	Indeno[1,2,3-cd] pyrene	4.17 ± 0.17 (mg/kg)
Polychlorinated biphenyl(PCB)	2,3,3′,4,4′- Pentachloro- biphenyl	19.6 ± 2.3 (μg/kg)	2,2′,4,4′,5,5′- Hexachloro- biphenyl	C40.0 ± 4.9 (μg/kg)
	2,3,3′,4′,6- Pentachloro -biphenyl	25.4 ± 1.9 (μg/kg)	2,2′,3,4′,5,5′,6- Heptachloro- biphenyl	17.1 ± 1.4 (μg/kg)
	2,2′,3,4′,5′,6- Hexachloro- biphenyl	38.9 ± 2.6 (μg/kg)	2,2′,3,3′,4,4′,5,5′- Octachloro- biphenyl	19.1 ± 2.2 (μg/kg)

## Abbreviations

UPM	Urban particulate matter
TRPV1	transient receptor potential vanilloid 1
PDTC	Ammonium pyrrolidinedithiocarbamate
AP-1	activator protein-1
NF-κB	nuclear factor kappa B
TNF-α	tumor necrosis factor-α
IL-8	interleukin-8
